# Part 1: The Role of Thyroglobulin Antibodies in Thyroid Cancer Development and Aggressiveness

**DOI:** 10.3390/cancers18050790

**Published:** 2026-02-28

**Authors:** Rodis D. Paparodis, Evangelos Karvounis, George Simeakis, Ioannis Androulakis, Dimitrios P. Askitis, Sarantis Livadas, Andreas Rizoulis, Vasileios Papanikos, Nicholas S. Mastronikolis, Dimitra Zianni, Charalampos Massouras, Ilias Perogamvros, Anastasios Boniakos, Nicholas G. Angelopoulos, Dimitra Bantouna, Aikaterini Kapezanou, Ourania Aporelli, Juan Carlos Jaume

**Affiliations:** 1 Hellenic Endocrine Network, 10563 Athens, Greecegsimeakis@gmail.com (G.S.);; 2 Endocrinology, Diabetes and Metabolism Clinics, Private Practice, 60141 Patras, Greece; 3 Division of Endocrinology, Diabetes and Metabolism, Department of Medicine, Loyola University Chicago, Edward Hines Jr. VA Hospital, Hines, IL 60141, USA; 4 Center of Excellence in Endocrine Surgery, Euroclinic Hospital, 11521 Athens, Greece; 5 Thyroid Neoplasia Clinic, Division of Endocrinology, Diabetes and Metabolism, 401 General Military Hospital, 15561 Cholargos, Greece; 6 Endocrinology, Diabetes and Metabolism Clinics, Private Practice, Chania, Greece; 7 Endocrinology, Diabetes and Metabolism Clinics, Private Practice, Alexandoupolis, Greece; 8 Endocrinology, Diabetes and Metabolism Clinics, Private Practice, Athens, Greece; 9 Athens Medical Center, 15125 Athens, Greece; 10 Endocrinology, Diabetes and Metabolism Clinics, Private Practice, Larisa, Greece; 11 Division of Endocrinology, Diabetes and Metabolism, Iaso General Hospital, 41005 Larisa, Greece; 12 Department of Otorhinolaryngology—Head and Neck Surgery, School of Medicine, University of Patras, 26504 Patras, Greece; 13 Endocrinology, Diabetes and Metabolism Clinics, Private Practice, Pagkrati, Attica, Greece; 14 Division of Diabetes, Endocrinology & Gastroenterology, University of Manchester, Manchester M13 9PL, UK; 15 Endocrinology, Diabetes and Metabolism Clinics, Private Practice, Petroupolis, Greece; 16 Endocrinology, Diabetes and Metabolism Clinics, Private Practice, Kavala, Greece

**Keywords:** thyroid cancer, autoimmunity, thyroglobulin antibodies

## Abstract

Introduction: Thyroglobulin antibodies (Tg-Abs) are often thought to be associated with thyroid autoimmunity, and as such, a link with thyroid cancer has been found in some, but not all, studies. Tg-Abs are also found in non-autoimmune thyroid-follicular destruction. Given these contradictory results, we designed the present study. Methods: We reviewed data from patients undergoing thyroidectomy for different indications in multiple centers across Greece and the US over 10 years. We compared the incidence of thyroid cancer and its features of tumor aggressiveness among patients with positive (≥30 IU/mL) and negative (<30 IU/mL) Tg-Abs titers and the Tg-Abs titers among patients with cancer, benign disease, and those with and without features of tumor aggressiveness. Furthermore, we performed multivariate and multiple regression analyses to identify if these effects were independently statistically significant. Results: We reviewed n = 9463 consecutive thyroidectomies and included n = 2873 subjects in the present work: n = 1537 with thyroid cancer and n = 1336 with benign disease. The incidence of thyroid cancer was significantly higher in preoperative Tg-Abs+ subjects, n = 273/638 (57.2%), compared with Tg-Abs− subjects, n = 1063/2235 (52.4%), OR 1.21, *p* < 0.05. Tumor size was larger, and capsular invasion and lymph node involvement were significantly more common in Tg-Abs+ subjects. Mean Tg-Abs were higher in subjects with tumors with extrathyroidal extension, capsular invasion, and lymph node involvement. On regression, Tg-Abs positivity was not associated with thyroid cancer incidence or aggressiveness, though, with the important exception of lymph node involvement (*p* < 0.001). Conclusions: Elevated preoperative thyroglobulin-antibody titers do not seem to affect the likelihood of developing thyroid cancer, even though they seem to be a surrogate for lymph node metastasis (as opposed to thyroid peroxidase antibodies, TPO-Abs). Further work is needed to clarify the interplay of this immune response on thyroid cancer metastatic behavior.

## Simple Summary

In this study, we investigated the validity of a blood test to predict thyroid cancer presence and aggressiveness. The blood test measures antibodies raised against a thyroid protein, thyroglobulin. The results suggest that testing for the presence of thyroglobulin antibodies predicts the likelihood of metastasis to lymph nodes, a feature of cancer aggressiveness.

## 1. Introduction

Thyroid cancer incidence was rising constantly for many years [1], until a plateau was recently reached [2]. This trend was attributed to the rapidly increasing use of point-of-care ultrasound and the enhanced detection of potentially indolent tumors [3], even though that rise was evident in tumors of all stages [1]. At the same time, a rise in thyroid autoimmunity has been observed [4], leading to a question on the potential association between autoimmunity prevalence and thyroid cancer. In fact, studies published as early as the 1950s implied that there is a link between these two conditions, based on data from surgical series [5]. Over the years, multiple studies evaluated that link, including several from our group [6], and noted that papillary thyroid cancer frequently coexisted with what appears to be a chronic form of lymphocytic thyroiditis. The largest effect was seen in patients who were euthyroid prior to their surgery, and especially those with none or low thyroid peroxidase (TPO) antibody titers [6,7]. This association is believed to represent a tumor evasion of the immune system strategy, given the potent cytotoxic responses produced by TPO in the thyroid microenvironment [7].

On the other hand, thyroglobulin antibodies (Tg-Abs) are often described as another marker of thyroid autoimmunity, but they seem less able to promote cytotoxicity [8] or lead to an equally significant thyroid failure when TPO antibodies are not concurrently elevated [9]. Despite that, multiple studies found a link between Tg-Abs and thyroid cancer [10], in addition to their well-established role as a post-operative tumor marker, where serum thyroglobulin measurement alone could be deceiving [11]. Some studies even suggest a role of Tg-Abs in predicting thyroid cancer aggressiveness [12], while others argue against it [2]. Our clinical observations tend to contradict the findings of most of these studies, since we seem to find that Tg-Abs do not predict the development of thyroid cancer or its aggressiveness. Therefore, we designed the present study to characterize any effect.

## 2. Materials and Methods

Our thyroid clinics, located in Greece and the US, are busy referral points where physicians provide comprehensive care for their patients with thyroid nodules. In these clinics, all agreeable patients are registered in prospectively collected registries, where clinical, laboratory, imaging, cytological, pathological, and treatment data are routinely recorded. In addition, patients who continue their follow-up in our outpatient clinics have data on tumor recurrence recorded as well.

For the present work, we retrieved data of patients undergoing thyroidectomy over 10 years from the University of Wisconsin Hospital Thyroid Multidisciplinary Clinic in Madison, WI; the Center of Excellence in Endocrine Surgery of the Euroclinic Hospital in Athens, Greece; the Division of ENT Surgery of the University of Patras, Greece; and 10 Hellenic Endocrine Network Endocrinology clinics scattered all over Greece. We included data from adult patients with a measurement of serum thyroglobulin antibodies within 3 months prior to the date of surgery. We excluded patients with no such measurements, as well as those with inadequate pathological data, those with a history of neck irradiation or exposure to ionizing radiation, those with mutations known to be directly related to thyroid cancer development (RET, RAS, pTEN, etc.), those with prior thyroid surgery, and those with cancers arising from cells other than follicular thyroid cells (medullary thyroid cancers, squamous cell carcinomas, lymphomas, metastatic/other cancers).

We collected data retrospectively on patients’ gender, age at surgery, preoperative Tg-Abs titers, TSH (when available), history of use of medications affecting the thyroid function (thyroid hormone supplementation/replacement or antithyroid medications), and surgical pathology. Both Tg-Abs and TSH were measured with commercially available radioimmunoassays at each study site.

Our subjects are grouped into two groups, based on their Tg-Abs titers: those with positive titers (Tg-Abs+), when Tg-Abs ≥ 30 IU/mL, and those with negative titers (Tg-Abs−), when Tg-Abs < 30 IU/mL. Our primary outcome is the difference in the incidence of thyroid cancer and the features of tumor aggressiveness between these two groups. Our secondary outcomes consist of [1] the difference in mean Tg-Abs concentration between subjects with benign and those with malignant disease, as well as [2] the difference in Tg-Abs concentration between subjects with thyroid cancer and features of tumor aggressiveness and those without these findings. Our tertiary outcome is the characterization of the potential independent role of high Tg-Abs in the incidence of thyroid cancer and its features of tumor aggressiveness.

Statistical analysis and graph generation were performed with GraphPad Prism v5.0 (GraphPad Software, Boston, MA, USA) and STATA v18.0 (StataCorp LLC, College Station, TX, USA). Categorical variables were compared with Fisher’s exact test or the χ^2^ test. Continuous variables were assessed for normality with the Kolmogorov–Smirnov test. When normality was not present, the data were log transformed, and normality was re-assessed. If the data did not follow the normal distribution either way, the non-parametric Kruskal–Wallis and Mann–Whitney tests were used, while the *t*-test and 1-way ANOVA were used for data following the normal distribution. For the tertiary outcome, multivariate and multiple logistic regression analyses were performed, and the coefficient B, standard error, z, *p* value, odds ratio, and 95% confidence intervals were calculated. In all analyses, *p* values < 0.05 were deemed significant.

## 3. Results

We reviewed n = 9463 consecutive thyroidectomies: n = 4277 subjects with thyroid cancer and n = 5186 subjects with benign histology. Out of these, we excluded n = 32 subjects with mutations that are known to directly produce thyroid cancers (see METHODS), n = 117 with prior thyroid surgery, n = 8 with a history of neck irradiation, and 6433 subjects who had no Tg-Abs measurements available in the last 3 months, prior to their thyroidectomy surgery. We ended up including in the present analysis n = 2873 subjects with complete data: n = 1537 with thyroid cancer and n = 1336 with benign disease. Out of these, n = 745 cases were recruited in the US (University of Wisconsin Hospital and Clinics, Madison, WI, USA) and n = 2128 in Greece (n = 1340 at the Euroclinic Hospital, Athens, Greece; n = 25 at the University of Patras Hospital ENT Department, Patras, Greece; and n = 763 at various Hellenic Endocrine Network referral centers).

### 3.1. Primary Outcome

**Figure 1:**
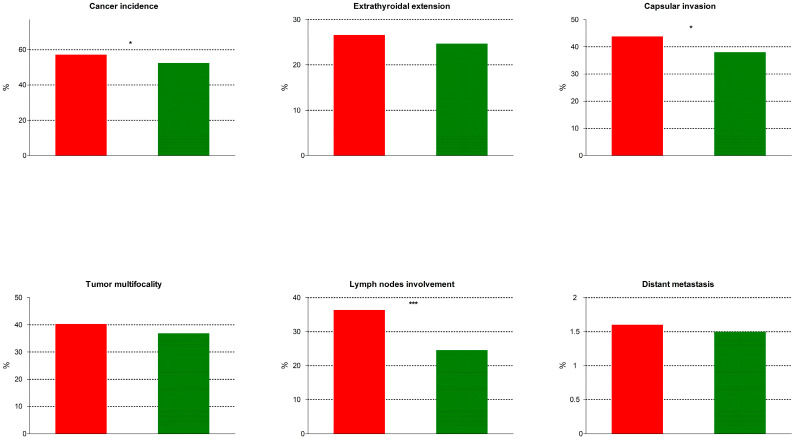
Positive thyroglobulin antibody titers (red bars) vs. negative (green bars). From left to right (**top panel**) cancer inci-dence; extrathyroidal extension; capsular invasion; (**bottom panel**) tumor multifocality; lymph node involvement; and distant metastasis. Asterisks indicate degrees of statistical significance.

The baseline characteristics of our population, including the two subgroups, along with their comparisons, are presented in Table 1. Overall, cancers were of the following histology: papillary thyroid cancer (PTC), n = 1494 (97.3%); follicular thyroid cancer (FTC), n = 35 (2.3%); anaplastic thyroid cancer (ATC), n = 1 (0.05%); and poorly differentiated thyroid cancer (PDTC), n = 7 (0.45%). Thyroid cancer incidence and the features of tumor aggressiveness and their comparisons are presented in Table 2 and depicted in Figure 1. In brief, thyroid cancer incidence was significantly higher in subjects with positive Tg-Abs titers, as were the rates of capsular invasion and lymph node involvement and the mean tumor size, compared to subjects with negative Tg-Abs.

### 3.2. Secondary Outcomes

**Figure 2:**
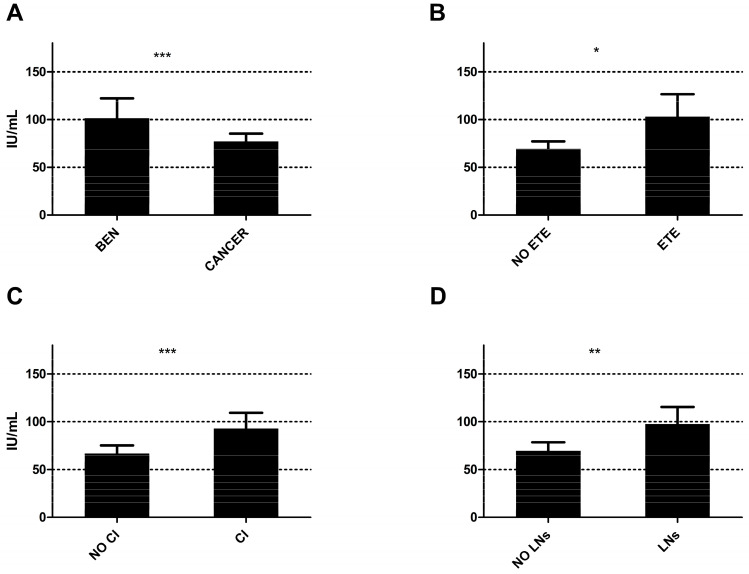
High (positive) Tg-Abs titers (≥30 IU/mL) vs. low (negative) Tg-Abs titers (<30 IU/mL) in (**A**) benign (BEN) and ma-lignant (CANCER) thyroid tumor subjects; (**B**) thyroid cancer subjects without (NO ETE) or with (ETE) extratyroidal extension; (**C**) without (NO CI) or with (CI) capsular invasion; and (**D**) without (NO LNs) or with (LNs) lymph node metastasis. Asterisks indicate degrees of statistical significance.

In our secondary analysis, we compared the Tg-Abs titers between patients’ groups with thyroid cancer and those with benign histology, as well as those with features of tumor aggressiveness and those without; the results are presented in Table 3 and depicted in Figure 2. In brief, subjects with thyroid cancer had lower Tg-Abs titers compared to those with benign disease, while subjects with extrathyroidal extension, capsular invasion, and lymph node metastasis had higher Tg-Abs titers compared to those who did not have these features. The mean Tg-Abs titers were significantly higher in subjects with macrocarcinomas (i.e., tumors > 1 cm in largest diameter) compared to those measured in subjects with microcarcinomas (i.e., tumors < 1 cm in largest diameter) (328.3 ± 1949.8 IU/mL vs. 61.6 ± 260.7 IU/mL, *p* < 0.0001).

### 3.3. Tertiary Outcomes

Our logistic regression analysis consisted of three steps: in the first step, we examined the influence of Tg-Abs as an independent predictor of thyroid cancer. The analysis showed that the univariate model was borderline insignificant (χ^2^(1) = 3.7, *p* = 0.053); the coefficient b was 0.2, indicating that if Tg-Abs are high (≥30 IU/mL), the probability of identifying cancer by surgical pathology increases insignificantly. The odds ratio of 0.8 means that the odds of identifying cancer by surgical pathology are 0.8 times as likely when Tg-Abs are high.

The second step assessed the effect of high Tg-Abs titers on the risk for thyroid cancer in a multivariate model, including as cofactors the subjects’ age, gender, country of origin, preoperative TSH, and the preoperative use of thyroxine, antithyroid medications, or the absence of treatment. This model was highly significant overall, with preoperative use of thyroxine and male gender being positively associated with the risk for cancer, and advanced age and the use of antithyroid medications being negatively associated with this outcome, but Tg-Abs were not independently associated with it (Table 4).

The third step assessed the effects of high Tg-Abs titers with features of tumor aggressiveness, such as tumor size, extrathyroidal extension, capsular invasion, lymph node involvement, and distant metastasis in multivariate analyses. The results are presented in Table 5. In brief, high Tg-Abs titers were not associated with any of these parameters, with the notable exception of lymph node involvement, where a strong, statistically significant association was found.

### 3.4. Ad Hoc Analysis of Macrocarcinomas Alone

An ad hoc analysis was performed after excluding all subjects with thyroid microcarcinomas (i.e., tumors < 1 cm in largest diameter, n = 750), leaving n = 787 subjects with thyroid macrocarcinomas (i.e., tumors > 1 cm in largest diameter): Tg-Abs+ n = 202 and Tg-Abs− n = 585. In this analysis, thyroid cancer incidence was statistically significantly higher in Tg-Abs+ subjects as compared to Tg-Abs− subjects [42.5% (202/475) vs. 35.5% (585/1648), odds ratio 1.35, 95% confidence interval 1.09–1.66, χ^2^ *p* = 0.005]. Extrathyroidal extension (Tg-Abs+ n = 64/202, 31.7% vs. Tg-Abs− n = 153/585, 26.2%, *p* = 0.28), capsular invasion (Tg-Abs+ n = 99/202, 49.0% vs. Tg-Abs− n = 248/585, 42.4%, *p* = 0.10), mean number of tumor foci (Tg-Abs+ 1.9 ± 1.3 vs. Tg-Abs− 1.9 ± 1.7, *p* = 0.32), multifocal tumors incidence (Tg-Abs+ n = 102/202, 50.5% vs. Tg-Abs− n = 270/585, 46.1%, *p* = 0.29) or distant metastasis (Tg-Abs+ n = 6/202, 3.0% vs. Tg-Abs− n = 13/585, 2.2%, *p* = 0.60) were not significantly different among the two groups. Lymph node involvement, however, was significantly more frequent in tumors arising in Tg-Abs+ subjects (Tg-Abs+ n = 87/202, 43.1% vs. Tg-Abs− n = 167/585, 28.5%, odds ratio 1.89, 95% confidence interval 1.36–2.64, *p* = 0.0002). Mean tumor size was significantly larger in Tg-Abs+ subjects (1.8 ± 1.0 cm) as compared to Tg-Abs− subjects (2.0 ± 1.2 cm), *p* = 0.025 as well.

### 3.5. Ad Hoc Analysis Using Higher Thyroglobulin Antibodies Titer Cutoff

Since some studies have used a different cutoff of 60 IU/mL to separate Tg-Abs negativity (Tg-Abs– < 60 IU/mL) and Tg-Abs positivity (Tg-Abs+ ≥ 60 IU/mL), another ad hoc analysis was performed using that cutoff in order to allow for the use of this dataset in future meta-analyses. In that analysis, the results were similar, since thyroid cancer incidence was higher in Tg-Abs+ subjects, n = 261/437 (59.7%), compared to Tg-Abs− subjects, n = 1276/2436 (52.4%) (odds ratio 1.35, 95% confidence interval 1.10–1.66, *p* = 0.005).

## 4. Discussion

Thyroid autoimmunity in the form of chronic lymphocytic thyroiditis has been linked to follicular-cell-derived thyroid cancers in surgical series, with multiple studies showing a strong association between these two entities [6,13]. Humoral autoimmune responses manifest themselves in the form of a high titer of thyroid autoantibodies, namely thyroid peroxidase antibodies and thyroglobulin antibodies. The first are mediators of categorical autoimmune humoral responses associated with thyroid parenchymal destruction, thereby leading to hypothyroidism and elimination of follicular cells [9]. In a cohort study from our group, high TPO titers were found to protect from thyroid cancer in an inverse linear relationship between their titers and thyroid cancer risk [7], while in a more recent study, we found that aggressive tumors tend to develop in those patients without any TPO antibodies in their serum ([14] and Part 2). Other studies from the Western world reported similar results, while multiple studies from China contradicted these findings [15].

On the other hand, thyroglobulin antibodies (Tg-Abs), although reportedly linked to an autoimmune response, are more rarely associated with hypothyroidism than TPO antibodies [16]. These antibody titers also seem related to the presence of follicular-cell derived thyroid cancers, especially papillary thyroid cancer (PTC), in several [17,18,19,20,21] but not all studies [22]. Out of these, two large retrospective studies from China found an independent association between elevated Tg-Abs titers and thyroid cancer in multivariate logistic regression analysis [17,19], even though rising titers did not produce stronger effects [19]. On the contrary, this association was no longer significant when TSH was accounted for in another Chinese study including 1400 patients that underwent thyroidectomy [20]. Some other studies claim that there is a correlation between Tg-Abs titers and features of tumor aggressiveness as well [12]. Specifically, Tg-Abs titers > 1150 IU/mL were linked to central lymph node metastasis in a multivariate analysis from a study of 214 patients with papillary thyroid cancer also from China [23]. Lymphatic invasion and lateral lymph node metastasis were found more commonly in patients with positive Tg-Abs in a study of 1171 patients with differentiated thyroid cancer from Korea as well [12], although some degree of bias could be present, since more lymph nodes were dissected in the Tg-Abs positive group in that study. Another study from China, including 2926 patients with PTC, suggested that higher Tg-Abs titers predict more indolent disease in the form of lower likelihood of extrathyroidal extension, and when combined with high TPO titers, lower incidence of central lymph node metastasis [17]. In concordance with these findings, an older study from Australia, including data from 1770 patients with differentiated thyroid cancer, found that Tg-Abs did not affect tumor aggressiveness or disease-related outcomes, such as overall or disease-free survival in multivariate analysis [24]. Additionally, patients with benign cytology by FNA who were operated on with total thyroidectomy were found to harbor thyroid cancers more frequently when thyroid autoantibodies (TPO and/or Tg-Abs titers) were elevated [25]. This association could be due to the effects of chronic lymphocytic thyroiditis inducing tumorigenesis of small, more commonly multifocal, but less aggressive thyroid tumors overall [26].

Our study is in agreement with most of these works regarding the effect of elevated Tg-Abs titers on thyroid cancer incidence *when that effect is estimated in a univariate fashion*, claiming that titers higher than 30 IU/mL raise the risk for thyroid cancer (Table 2). This was also evident in the ad hoc analyses, when macrocarcinomas only were assessed, as well as when using a higher cutoff of 60 IU/mL of Tg-Abs titers. This does not seem to happen, though, when a comprehensive multivariate model is used, where gender, age, country of origin, use of thyroid hormone affecting medications, and preoperative serum TSH are all accounted for (Table 4). It is impressive to see, though, the strong positive effects of younger age, male gender, and use of thyroxine supplements, in that risk, as well as the protective effects of the use of antithyroid medications, which are an indirect marker of preoperative hyperthyroidism (Table 4), proving that our results are in concordance with global literature overall.

Furthermore, our study found higher Tg-Abs titers in patients harboring tumors with extrathyroidal extension, capsular invasion, or lymph node involvement, as compared to those without these features, while the univariate analysis identified a statistically significant effect with regard to capsular invasion, tumor size, and lymph node involvement as well (Table 3). Even though these findings are in agreement with some of the previously mentioned studies, the multivariate regression analysis failed to confirm these associations, with the notable exception of the presence of lymph node involvement (Table 5).

The cancer immunoediting hypothesis [27] suggests that “escape” mechanisms enable metastatic cancer cells (in our case, follicular thyroid cancer cells) to migrate to draining lymph nodes. It is well established [28] that thyroglobulin antibodies (Tg-Abs) are not produced within the thyroid gland itself but arise from B cells in lymphoid tissues. These B cells are activated by thyroglobulin either leaking from the gland or, particularly in the case of metastatic thyroid cancer, by thyroglobulin-expressing malignant cells present in lymph nodes. Thus, it is more than speculative to conclude that metastatic thyroid cancer cells producing thyroglobulin in lymph nodes are positioned at the optimal anatomical site for Tg-Ab production. Furthermore, unlike TPO, a stable membrane-bound protein [29], soluble thyroglobulin inside the thyroid follicle exists as a highly ordered dimeric quaternary structure stabilized by the ionic environment of the colloid. Once outside this environment, its quaternary structure likely becomes disrupted, potentially exposing neo-epitopes and triggering an immune response. Therefore, thyroglobulin in lymph nodes is a probable source of immunogenicity and Tg-Ab production.

### 4.1. Strengths of the Study

Our study is the largest study to date (to our knowledge) originating from the Western world assessing the effects of elevated preoperative thyroglobulin antibody titers on the risk of thyroid cancer, and its results are consistent with findings from other studies from the Western world [24]. Additionally, it is the only study published to date to incorporate data from patients operated on in multiple centers in more than one country. Furthermore, it is the first study to account for the preoperative use of medications affecting thyroid hormone concentrations, in addition to accounting for preoperative serum TSH, confirming our previously published findings on the absence of a role of TSH in thyroid cancer development in the presence of thyroid autoimmunity [30].

### 4.2. Limitations of the Study

Similar to all previously published studies of this kind, our study is limited by multiple factors, the most important being its retrospective nature, even though our data are collected in a prospective manner. Secondly, our thyroglobulin antibody measurements are performed in multiple, different laboratories, sharing different reagents and potentially different reference ranges, even though they were all performed in well-established laboratories using well-validated radioimmunoassays. A third limiting factor is the absence of preoperative serum Tg-Abs titers measurements in a large proportion of the patients operated on in our centers (which is not a standard of care) [31,32,33]. Also, Tg-Abs titers are known to have significant fluctuations over time, even though these measurements were all performed within 3 months prior to surgery in all subjects. In addition, the use of data from mostly Caucasian patients limits the generalizability of our results to other populations, especially Asians, where the results from multiple other studies differ significantly [17,19,23]. This has been the case with our prior work on TPO antibodies [7], but other studies in Caucasian populations were in agreement with our findings as well [15]. Lastly, surgical pathology data originate from multiple practices, which might not follow the exact same reporting style/methods, thus allowing for some bias, even though this increases the generalizability of our results.

## 5. Conclusions

Elevated preoperative thyroglobulin antibody titers constitute a mediocre preoperative marker of increased risk of thyroid cancer but a solid predictor of lymph node metastasis. Tg-Abs positivity remains independently associated with lymph node metastasis but not with overall cancer incidence or other aggressiveness features. Therefore, we recommend their measurement as part of the preoperative endocrine evaluation in all patients planned to undergo surgery for potentially malignant thyroid nodules.

Overall, their role in the immune response, their relationship with thyroid cancer, and their potential to enhance or suppress tumorigenesis remain controversial and warrant further exploration with studies assessing their potential effects in the tumor and lymph node immune microenvironment ([34] and Part 2).

## Figures and Tables

**Table 1:** Baseline characteristics of the study population and comparison between those with positive and negative Tg-Abs.

	Entire Population	Tg-Abs+ ≥30 IU/mL	Tg-Abs– <30 IU/mL	*p* Value
**Males n (%)**	750 (26.1%)	140 (21.9%)	610 (27.3%)	**0.008**
**Females n (%)**	2123 (73.9%)	498 (78.1%)	1625 (72.7%)	
**Age, mean (SD)**	47.0 (14.5)	45.7 (14.8)	47.4 (14.4)	**0.019**
**TSH, mean (SD)**	2.11 (5.09)	2.36 (4.31)	2.04 (5.29)	**0.001**
**TSH in non-users, mean (SD)**	2.14 (5.21)	2.35 (3.23)	2.03 (5.46)	**<0.0001**
**LT4 n (%)**	585 (20.4)	220 (34.5)	365 (16.3)	**<0.0001**
**AT n (%)**	124 (4.3)	46 (7.2)	78 (3.5)	
**NONE n (%)**	2164 (75.3)	372 (58.3)	1792 (80.2)	
**Tg-Abs titer, mean (SD)**	96.0 (614.6)	369.2 (1162.6)	8.7 (8.0)	**<0.0001**

Legend: SD: standard deviation; non-users: subjects who were not receiving any medication affecting thyroid hormone concentrations prior to thyroid surgery; LT4: number of subjects receiving thyroid hormone supplementation/replacement therapy; AT: number of subjects receiving antithyroid medications; NONE: number of subjects not on any thyroid function-related treatment.

**Table 2:** Thyroid cancer incidence and features of tumor aggressiveness in the entire study population and each subgroup, along with their comparisons.

	Entire Population (n)	Tg-Abs+ ≥30 IU/mL	Tg-Abs– <30 IU/mL	*p* Value	Odds Ratio (95% CI)
**Benign n (%)**	1336	273/638 (42.8%)	1063/2235 (47.6%)	**0.037**	1.21 (1.02–1.45)
**Cancer n (%)**	1537	365/638 (57.2%)	1172/2235 (52.4%)		
**PTC n (%)**	1494/1537	359/365 (98.4%)	1135/1172 (96.8%)	0.15	0.51 (0.21–1.23)
**FTC n (%)**	35/1537	5/365 (1.4%)	30/1172 (2.6%)	0.23	0.53 (0.20–1.37)
**ATC/PDTC n (%)**	8/1537	1/365 (0.3%)	7/1172 (0.6%)	0.69	0.46 (0.06–3.73)
**ETE n (%)**	386/1537	97/365 (26.6%)	289/1172 (24.7%)	0.49	1.11 (0.85–1.45)
**CI n (%)**	605/1537	160/365 (43.8%)	445/1172 (38.0%)	**0.0496**	1.28 (1.01–1.62)
**LNs n (%)**	421/1537	133/365 (36.4%)	288/1172 (24.6%)	**<0.0001**	1.76 (1.37–2.26)
**METs n (%)**	23/1537	6/365 (1.6%)	17/1172 (1.5%)	0.81	1.14 (0.44–2.90)
**Tumor size (cm)** **Mean (SD)**	1.5 (2.0)	1.8 (2.7)	1.4 (1.7)	**0.006**	.
**Tumor foci** **Mean (SD)**	1.8 (1.9)	1.9 (1.6)	1.8 (1.9)	0.09	.
**Multifocal n (%)**	580/1537 (37.7%)	147/365 (40.3%)	433/1172 (36.9%)	0.27	1.15 (0.90–1.46)
**Macrocarcinoma** **n (%)**	787/1537 (51.2%)	202/365 (55.3%)	585/1172 (49.9%)	0.08	1.24 (0.97–1.56)

Legend: PTC: papillary thyroid cancer; FTC: follicular thyroid cancer; ATC: anaplastic thyroid cancer; PDTC: poorly differentiated thyroid cancer; ETE: extrathyroidal extension; CI: capsular invasion; LNs: lymph node involvement; METs: distant metastasis; SD: standard deviation. The percentage rates of the histological types of cancers and the aggressive features refer to the total number of cancers of the group.

**Table 3:** Mean Tg-Abs (IU/mL) titers in different subjects’ subgroups and their comparisons.

	Presence of Feature	Absence of Feature	*p* Value
**Thyroid Cancer** **Mean (SD)**	77.2 (318.0)	.	**0.0001**
**Benign Histology** **Mean (SD)**	101.2 (757.7)	.	
**PTC** **Mean (SD)**	79.0 (323.0)	.	0.11
**FTC** **Mean (SD)**	20.2 (54.5)	.	
**ATC/PDTC** **Mean (SD)**	17.8 (18.5)	.	
**ETE** **Mean (SD)**	103.0 (443.4)	69.4 (268.7)	**0.02**
**CI** **Mean (SD)**	92.9 (401.3)	66.9 (248.4)	**0.0004**
**LNs** **Mean (SD)**	97.5 (367.8)	69.4 (477.8)	**0.002**
**Multifocality** **Mean (SD)**	58.2 (180.5)	88.8 (377.9)	0.89
**METs** **Mean (SD)**	264.5 (650.8)	74.3 (309.5)	0.23

Legend: PTC: papillary thyroid cancer; FTC: follicular thyroid cancer; ATC: anaplastic thyroid cancer; PDTC: poorly differentiated thyroid cancer; ETE: extrathyroidal extension; CI: capsular invasion; LNs: lymph node involvement; METs: distant metastasis; SD: standard deviation.

**Table 4:** Multivariate logistic regression analysis estimating the effect of high Tg-Abs titers on the risk for thyroid cancer.

Variable	Coefficient	Std. Error	Wald	*p* Value
**Age**	−0.03	0.003	72.56	**<0.0001**
**Country = USA**	−0.27	0.16	2.76	0.097
**Male gender**	0.22	0/10	4.54	**0.0331**
**Preop Rx = AT**	−0.74	0.21	12.64	**0.0004**
**Preop Rx = LT4**	0.43	0.11	15.83	**0.0001**
**Preoperative TSH**	0.01	0.01	1.01	0.31
**High Tg-Abs**	0.11	0.11	0.99	0.32
**Constant**	1.46	0.17	72.54	**<0.0001**

Legend: Country of origin of the subjects; Rx: preoperative treatment with medications affecting thyroid function; LT4: levothyroxine; AT: antithyroid medications; High Tg-Abs: Tg-Abs titers ≥ 30 IU/mL.

**Table 5:** Multivariate analyses results on the effects of high Tg-Abs titers on the features of thyroid cancer tumor aggressiveness. All analyses are multivariate logistic regression analyses, including as cofactors the subjects’ age, gender, country of surgery, TSH, and use of medications affecting thyroid hormone concentrations.

Variables	Coefficient	Standard Error	*p* Value
**Tumor size**	−0.2	0.6	0.75
**ETE**	0.2	0.2	0.21
**CI**	0.1	0.1	0.67
**LNs**	0.6	0.1	**<0.001**
**METs**	0.5	0.5	0.37

Legend: ETE: extrathyroidal extension; CI: capsular invasion; LNs: lymph node involvement; METs: distant metastasis.

## Data Availability

The original contributions presented in this study are included in the article. Further inquiries can be directed to the corresponding authors.
